# Comparative Biochemical Profiling of Three Commercial Date Palm Cultivars (Sukkary, Mejhoul, and Khlass) During Fruit Development

**DOI:** 10.3390/ijms27167152

**Published:** 2026-08-10

**Authors:** Naoki Terada, Nasratullah Habibi, Abdelazize Eljiati, Abdelgawwad Ali Abbady Gadelkarim, Atsushi Sanada, Atsushi Kamata, Kaihei Koshio

**Affiliations:** 1 Graduate School of Agriculture, Tokyo University of Agriculture, 1-1-1 Sakuragaoka, Setagaya-ku, Tokyo 156-8502, Japan; nt204361@nodai.ac.jp (N.T.); a3sanada@nodai.ac.jp (A.S.); koshio@nodai.ac.jp (K.K.); 2 Faculty of Agriculture, Balkh University, Balkh 1701, Afghanistan; 3 National Center for Palms & Dates (NCPD), Signature Building, Al Dahi, Hittin, Riyadh 13512, Saudi Arabia; a.eljiati@gmail.com; 4 Research and Development Department, Yousef Bin Abdul Latif and Sons Agriculture Co., Ltd., Unaizah 52758, Saudi Arabia; a.gadelkarim@yaljgroup.com; 5 Faculty of Agriculture, Kurokawa Field Science Center, Meiji University, 2060-1 Kurokawa, Aso-ku, Kawasaki-shi 215-0035, Japan; kamataatsushi@meiji.ac.jp

**Keywords:** date palm (*Phoenix dactylifera* L.), organic acids, sugars, Sukkary, Mejhoul, Khlass

## Abstract

Date palm (*Phoenix dactylifera* L.) plays a vital agronomic and nutritional role in arid regions of the Gulf Cooperation Council (GCC), where sustainable crop improvement is essential. Among the many cultivars, Sukkary, Mejhoul, and Khlass stand out for their commercial importance, adaptability, and distinctive fruit characteristics. This study investigated their metabolomic profiles during fruit development, focusing on sugars, amino acids, polyphenols, and organic acids. Distinct biochemical signatures were identified among cultivars. Mejhoul exhibited the highest Brix (~79), reflecting elevated glucose and fructose, while Sukkary and Khlass (~72) showed sucrose predominance. Sugar profiles revealed cultivar-specific patterns: Sukkary accumulated more sucrose, whereas Mejhoul and Khlass had higher invert sugars, with inositol uniquely abundant in Khlass. Amino acid analysis showed Sukkary enriched in glutamine, valine, and GABA—metabolites linked to nitrogen metabolism and stress tolerance. Mejhoul contained more tyrosine and glutamine, and Khlass was rich in methionine and urea. Organic acid profiling highlighted Sukkary’s dominance in TCA intermediates (fumaric, succinic, and malic acids), Khlass in citric and citraconic acids, and lower organic acid levels in Mejhoul. Sukkary also exhibited the highest polyphenol content, consistent with elevated phenylalanine and activation of the phenylpropanoid pathway. These metabolomic distinctions reflect cultivar-specific physiological and nutritional traits: Mejhoul suits fresh consumption and fermentation, Sukkary favors functional food and storage potential, and Khlass offers balanced sugar-acid and inositol profiles. The findings provide a biochemical basis for cultivar-specific breeding, postharvest optimization, and enhanced nutritional value under climate stress.

## 1. Introduction

The date palm (*Phoenix dactylifera* L.) is one of the key tree species for agriculture in the semi-arid and arid regions of the Middle East and North Africa (MENA). It is now cultivated in South Asia, South Africa, Iberia, the Americas, and Australia [1]. In 2024, world production of dates was estimated at about 9.9 million tonnes, with cultivation on over 1 million hectares [2]. The Gulf Cooperation Council (GCC) countries—Bahrain, Kuwait, Oman, Qatar, Saudi Arabia, and the United Arab Emirates—collectively encompass about 259 million hectares characterized by arid and semi-arid conditions. These environments are marked by extremely low and unpredictable rainfall, intense heat, fragile and erosion-prone soils, and severe land degradation, with desertification affecting more than 95% of the region [3]. Despite these severe environmental constraints, agricultural development in the GCC has advanced through the strategic use of groundwater, desalinated water, and treated wastewater. Strong government support has also been instrumental in driving improvements in crop productivity and promoting more sustainable farming practices [4]. Among the crops capable of thriving in such harsh environments, the date palm (*Phoenix dactylifera* L.) stands out for its remarkable historical, cultural, ecological, and economic significance across the Middle East, the Mediterranean region, and North Africa [4,5,6]. Cultivated for thousands of years, the date palm played a foundational role in ancient civilizations. Archeological evidence—including coins dating back to 485 BC bearing palm imagery—demonstrates its early domestication and its adaptation to arid environments. The high cultivar diversity observed in the Western Mediterranean further suggests that date palms underwent multiple domestication events [6].

Belonging to the Arecaceae family, *Phoenix dactylifera* is one of 14 species within the Phoenix genus, which is native to tropical and subtropical regions of southern Asia and Africa [7,8,9,10]. The trees can attain impressive heights of 21–23 m, forming tall, sturdy trunks topped with a dense crown of foliage. Their arching fronds, which can reach up to 6 m in length, create a distinctive canopy structure that not only defines the tree’s appearance but also contributes to its efficiency in capturing sunlight and withstanding harsh desert conditions [1,11]. Globally, more than 100 million date palms are cultivated, with the greatest concentrations found across the Middle East. Significant production also occurs in regions such as Australia, Mexico, South America, southern Africa, and the United States, reflecting the crop’s adaptability to warm, dry climates and its growing economic importance worldwide [12]. The fruit undergoes four distinct ripening stages—Kimri, Khalal, Rutab, and Tamer—each marked by characteristic changes in color, texture, and taste. As the fruit progresses through these stages, its biochemical and nutritional profile evolves, with the edible mesocarp becoming increasingly rich in sugars, dietary fiber, and a diverse array of antioxidants. These transformations contribute to the unique sensory qualities and health benefits associated with dates at different stages of maturity [13,14,15,16].

Beyond their agronomic importance [17,18], date fruits are highly valued for their exceptional nutritional composition and health-promoting properties [19,20,21,22]. They are a rich source of natural sugars, providing readily available energy, and contain significant amounts of dietary fiber, which supports digestive health. Dates are also packed with essential vitamins, such as B-complex vitamins and vitamin K, as well as minerals including potassium, magnesium, and iron [23,24]. Moreover, they are abundant in bioactive compounds with strong antioxidant activity, such as flavonoids, phenolic acids, and carotenoids, which help protect cells from oxidative stress, reduce inflammation, and contribute to overall health and disease prevention [23,24,25]. Tunisian date cultivars, including Gondi, Gasbi, Khalt Dhahbi, and Rutab Ahmar, are notable for their rich biochemical composition, particularly in phenolic compounds that contribute to their antioxidant potential and health benefits. During the Biser stage of fruit development, these cultivars exhibit especially high concentrations of key phenolics such as caffeic acid, ferulic acid, protocatechuic acid, and catechin. These compounds play a crucial role in neutralizing free radicals, thereby protecting cells from oxidative damage and reducing the risk of chronic diseases. The elevated phenolic content at this stage also influences the fruit’s flavor profile, color, and overall nutritional quality, making Tunisian dates not only a valuable dietary source of energy but also a functional food with significant bioactive properties [26,27,28]. These bioactive compounds offer substantial antioxidant potential, reinforcing the date fruit’s value in health foods and pharmaceutical applications [29].

Biochemical profiling is an essential tool for elucidating the complex composition and variability of date fruits across different developmental stages and environmental conditions. By analyzing key metabolites—such as sugars, organic acids, amino acids, vitamins, minerals, and bioactive compounds like phenolics and flavonoids—researchers can gain insights into how fruit quality, nutritional value, and antioxidant potential change as the fruit matures. This profiling also helps identify cultivar-specific biochemical traits and their responses to abiotic stresses, such as drought, salinity, or temperature fluctuations. Ultimately, comprehensive biochemical characterization informs breeding programs, guides postharvest handling strategies, and supports the development of dates as functional foods with targeted health benefits [30,31]. By profiling primary and secondary metabolites, including sugars, organic acids, amino acids, flavonoids, and phenolic compounds, metabolomics provides a comprehensive understanding of the biochemical complexity of date fruits. Sugars, such as glucose, fructose, and sucrose, constitute the main energy source and largely determine the sweetness and caloric content of the fruit. Organic acids, including citric, malic, and tartaric acids, contribute to the fruit’s flavor balance and influence pH and preservation characteristics. Amino acids, such as proline, glutamic acid, and aspartic acid, play roles in protein synthesis and stress responses, while flavonoids and phenolic compounds—including catechins, ferulic acid, and caffeic acid—exhibit strong antioxidant activity, helping to neutralize free radicals and reduce oxidative stress. Together, these metabolites define the nutritional quality, sensory properties, and potential health-promoting effects of different date cultivars, making metabolomic profiling a crucial tool for cultivar selection, breeding, and the development of functional foods [12,32]. Furthermore, metabolomic data helps elucidate plant physiological responses to abiotic stresses, support post-harvest quality monitoring, and assist in authenticating and differentiating cultivars for breeding and commercial purposes [33,34,35,36]. In the context of climate-resilient agriculture and functional food development, metabolomic profiling of dates represents a powerful tool for optimizing crop value chains and enhancing the health-promoting attributes of this traditional yet globally relevant fruit.

Sukkary, Khlass, and Mejhoul are among the most commercially important and widely cultivated date palm cultivars, each possessing distinct fruit characteristics and market value. Sukkary, predominantly cultivated in Saudi Arabia, is highly prized for its exceptionally soft texture, golden color, and high sugar content, making it one of the most popular premium dessert dates [37]. Khlass is recognized for its balanced sweetness, caramel-like flavor, and versatility for both fresh consumption and processing, and it is extensively cultivated throughout the Arabian Gulf region [38]. Mejhoul, originally associated with Morocco and now widely grown in several countries, is internationally regarded as a premium cultivar due to its large fruit size, rich flavor, and excellent postharvest quality, commanding a high value in global markets. These cultivars also differ in their fruit development patterns, biochemical composition, and sensory characteristics, making them ideal candidates for comparative metabolomic investigations aimed at understanding cultivar-specific metabolic variation during fruit maturation.

This study investigates the metabolomic profiles of three prominent date palm cultivars, Sukkary, Khlass, and Mejhoul, which are widely cultivated and valued for their fruit quality, yield, and market demand. Metabolomic profiling of these cultivars is essential for uncovering their unique biochemical signatures, which underpin their nutritional, therapeutic, and commercial value. Such profiling provides crucial insights into metabolic diversity, supports the development of functional foods, and guides breeding programs aimed at improving resilience, quality, and adaptation to environmental stress. The findings will support ongoing efforts in sustainable agriculture, health-focused food innovation, and genetic improvement of crops suited to some of the world’s most challenging agricultural landscapes.

## 2. Results

### 2.1. Comparison of Total Sugars (Brix)

**Figure 1:**
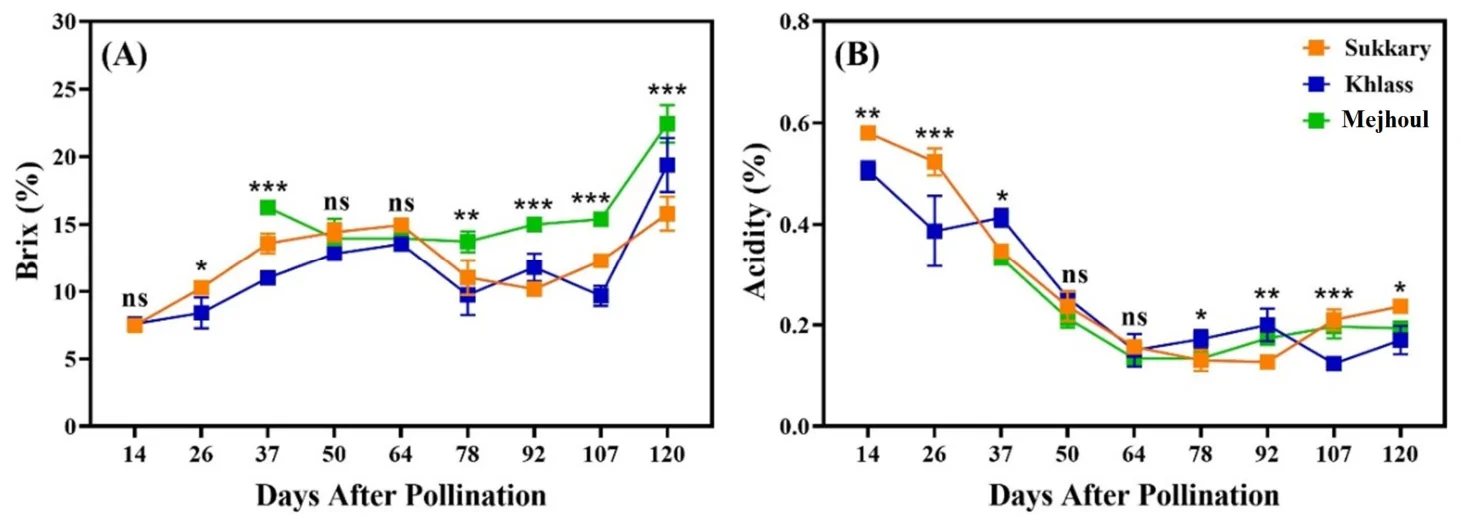
Temporal changes in total soluble solids (Brix %) and acidity in Sukkary, Khlass, and Mejhoul date palm cultivars during fruit development. Panel (**A**) shows the progression of Brix values. Panel (**B**) displays the corresponding changes in acidity. Values represent the mean of three biological replicates; error bars indicate standard deviation. Statistical significance is denoted as follows: *** *p* < 0.001, ** *p* < 0.005, * *p* < 0.01, ns = not significant.

The analysis revealed that all three date palm cultivars—Sukkary, Khlass, and Mejhoul—exhibited Brix values exceeding 5 at 14 days after pollination (DAP). The lowest Brix levels were observed at 14 DAP for Sukkary and Khlass, and at 37 DAP for Mejhoul. In contrast, the highest Brix content was recorded at 120 DAP across all cultivars (Figure 1A). Among the three, Mejhoul demonstrated significantly higher Brix values compared to Sukkary and Khlass throughout the observation period. Acidity content displayed a general decreasing trend over time. The highest acidity was recorded at 14 DAP for Sukkary and Khlass, whereas Mejhoul reached its peak acidity at 37 DAP. The lowest acidity values were observed at 64 DAP in all cultivars. At 120 DAP, Sukkary maintained significantly higher acidity compared to Khlass and Mejhoul (Figure 1B).

### 2.2. Sugar Content

**Figure 2:**
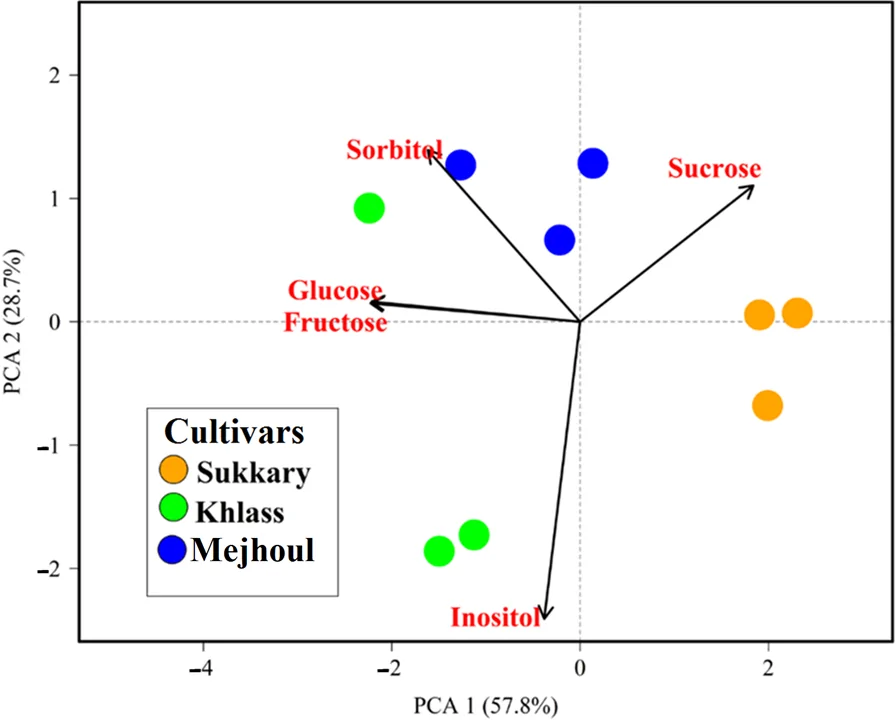
Principal component analysis (PCA) of sugar composition in Sukkary, Khlass, and Mejhoul date palm cultivars. PCA reveals strong clustering of cultivars according to dominant sugar profiles.

Principal component analysis (PCA) of sugar composition revealed clear distinctions among the three date palm cultivars based on their dominant sugar types. The first two principal components, PC1 and PC2, accounted for 57.8% and 28.7% of the total variance, respectively, indicating a strong differentiation in sugar profiles. Sucrose was the predominant sugar in the Sukkary cultivar, suggesting lower invertase activity and a preference for disaccharide accumulation during fruit development. In contrast, the Mejhoul and Khlass cultivars showed higher levels of the monosaccharides glucose and fructose, indicative of active sucrose hydrolysis and a more advanced ripening process. In the case of Khlass, inositol accumulation was found to be specific, which might well contribute to preventing metabolic syndromes. These differences reflect distinct cultivar-specific sugar metabolism pathways and developmental stages, which could influence fruit sweetness, texture, and market preference (Figure 2).

### 2.3. Changes in Sugar Content

**Figure 3:**
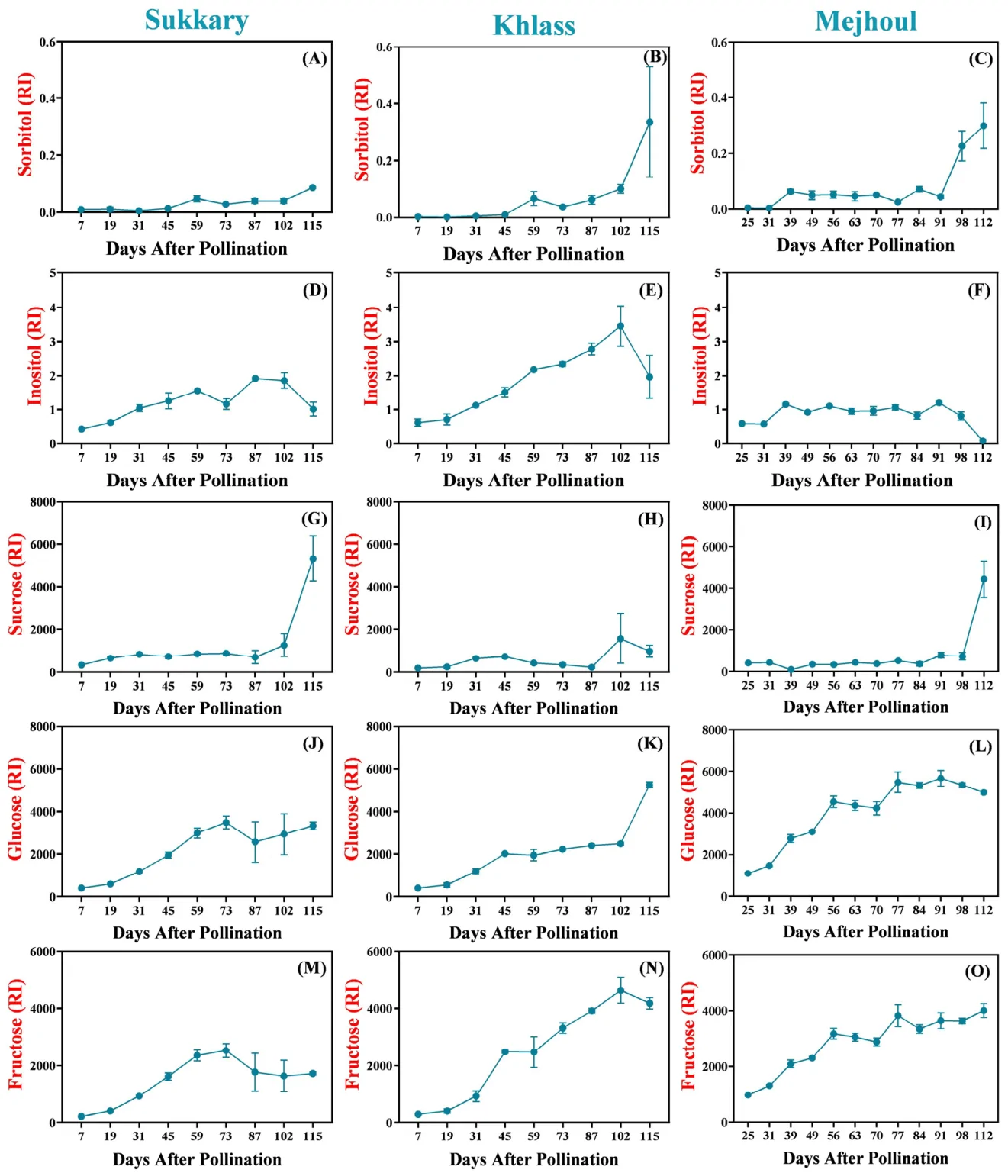
Temporal changes in the content of sugars in Sukkary, Khlass, and Mejhoul cultivars throughout fruit development. (**A**–**O**) Track changes in sorbitol, inositol, sucrose, glucose, and fructose from early development to maturity.

The analysis of sugar content revealed a general increasing trend in all three cultivars, Sukkary, Khlass, and Mejhoul, throughout the fruit development period. In Sukkary and Khlass, the highest sorbitol was observed at 115 DAP (Figure 3A,B), whereas in Mejhoul, the highest sorbitol was observed at 112 DAP (Figure 3C). The lowest sorbitol levels were detected at 31 DAP in Sukkary (Figure 3A), 7, 19, and 31 DAP in Khlass (Figure 3B), and at 25 DAP in Mejhoul (Figure 3C). Mejhoul exhibited higher sorbitol levels, with concentrations approximately 1.5 times higher than Khlass and 1.9 times higher than Sukkary. Inositol content followed distinct patterns among cultivars (Figure 3D–F). In Sukkary, the highest level of inositol was observed at 87 DAP, while the minimum was at 7 DAP (Figure 3D). In Khlass, the maximum and minimum inositol were measured at 102 DAP and 7 DAP, respectively (Figure 3E). In Mejhoul, the highest inositol level was recorded at 90 DAP, while the lowest was at 112 DAP (Figure 3F). Sucrose levels increased notably during fruit maturation. Sukkary showed a continuous rise in sucrose, with the highest level recorded at the final observation (115 DAP) and the lowest at 7 DAP (Figure 3G). In Khlass, the highest sucrose was recorded at 102 DAP, while the lowest was at 7 DAP and 87 DAP (Figure 3H). In Mejhoul, sucrose peaked at 112 DAP and was lowest at 39 DAP (Figure 3I). Among the three cultivars, Sukkary had the highest overall sucrose content, suggesting it is the dominant sugar in this variety (Figure 3G). Glucose content also varied among cultivars. Sukkary reached peak glucose levels at 73 DAP, with the lowest at 7 DAP (Figure 3J). Khlass showed a similar trend, peaking at 102 DAP and reaching its minimum at 7 DAP (Figure 3K). In Mejhoul, the highest glucose concentrations were recorded at 77 and 91 DAP, while the lowest occurred at 25 DAP (Figure 3L). At the final observation point, Mejhoul exhibited higher glucose content than Sukkary and Khlass. Fructose levels mirrored those of glucose, where in Sukkary, the peak was at 73 DAP, while the minimum was at 7 DAP (Figure 3M); Khlass displayed peak fructose levels at 102 and 115 DAP and the lowest at 7 DAP (Figure 3N), and Mejhoul showed the highest concentration at 115 DAP and the lowest at 25 DAP (Figure 3O). In the final observation, Mejhoul had accumulated more fructose than Sukkary and Khlass.

**Figure 4:**
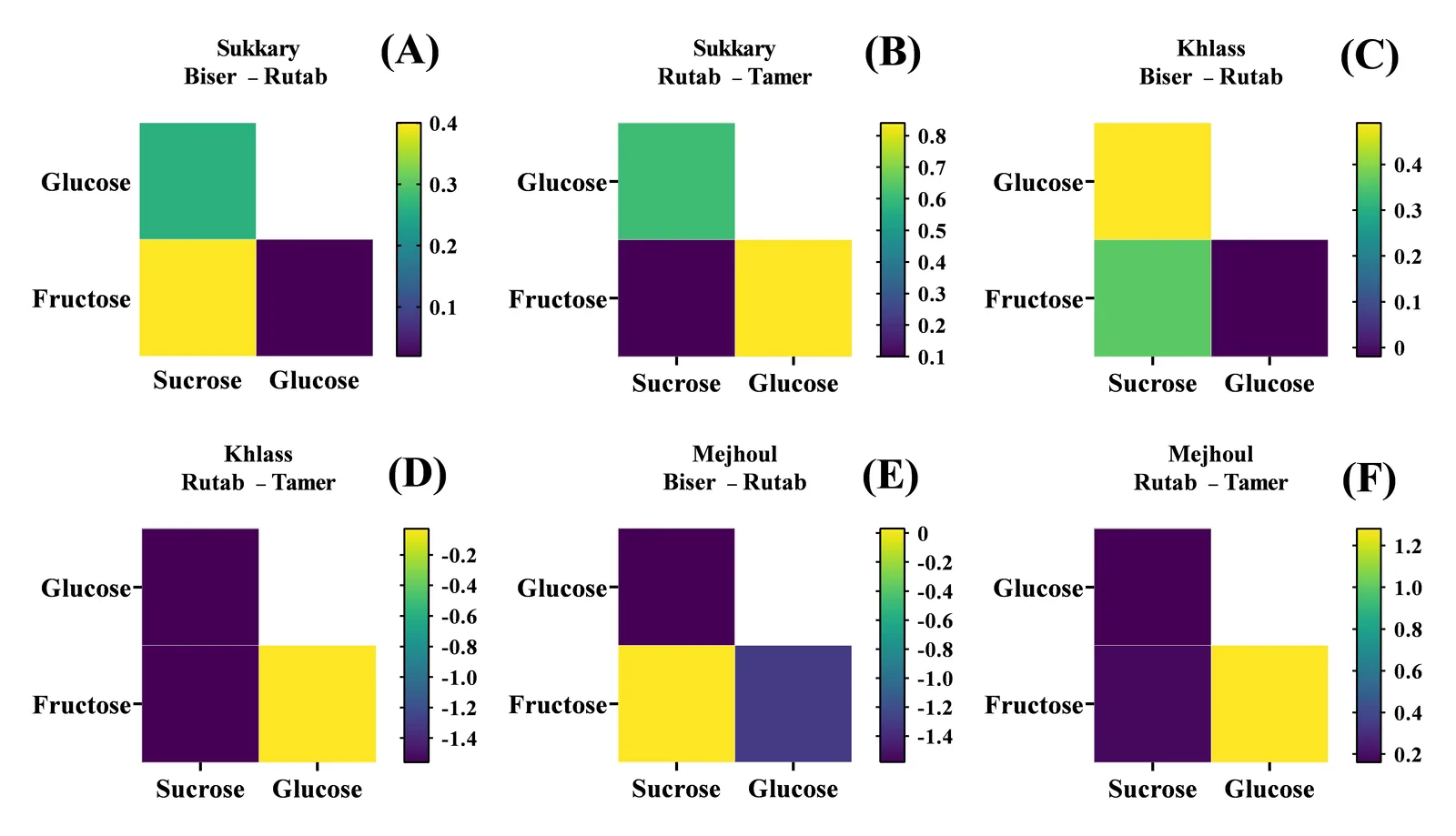
Changes in sugar content of Sukkary (**A**,**B**), Khlass (**C**,**D**), and Mejhoul (**E**,**F**) cultivars at the Biser to Rutab and Rutab to Tamer stages, respectively.

Sukkary showed greater glucose accumulation during the final ripening stage, Khlass displayed a shift from glucose accumulation at the Rutab stage to fructose dominance at the Tamer stage, and Mejhoul consistently accumulated fructose throughout ripening. These differences highlight genotype-specific regulation of carbohydrate metabolism, which likely contributes to the distinctive sweetness, flavor, and fruit quality characteristics of each date palm variety (Figure 4).

### 2.4. Organic Acids Content

**Figure 5:**
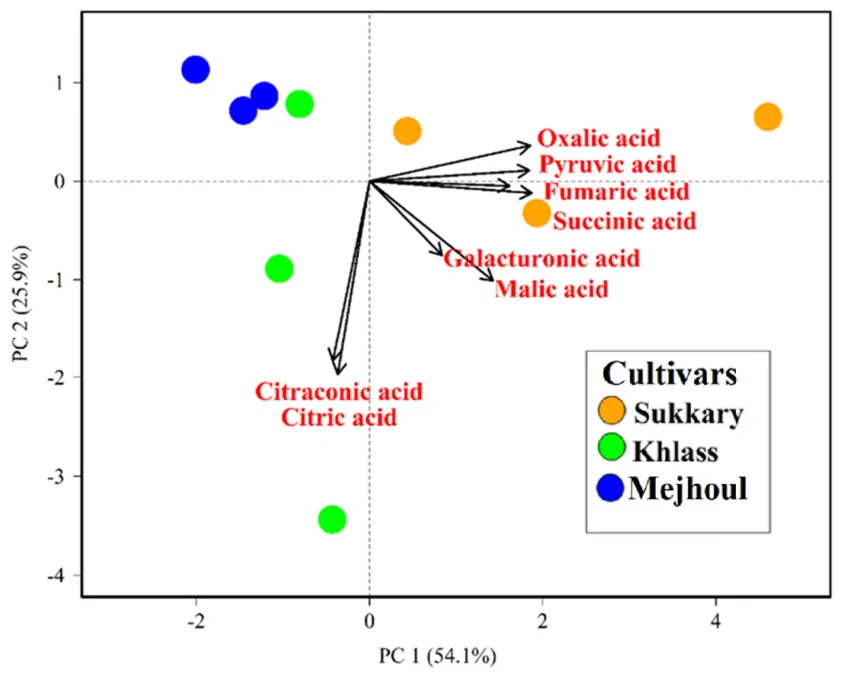
Principal component analysis (PCA) of organic acid profiles in Sukkary, Khlass, and Mejhoul date palm cultivars.

Principal component analysis (PCA) of organic acid profiles demonstrated distinct metabolic signatures among the three date palm cultivars, with PC1 accounting for 54.1% and PC2 for 25.9% of the total variation (cumulative 80.0%). Sukkary was associated with elevated levels of oxalic acid, pyruvic acid, fumaric acid, succinic acid, galacturonic acid, and malic acid, suggesting enhanced activity in both the tricarboxylic acid (TCA) cycle and cell wall metabolism. These organic acids are often linked to fruit maturation, respiration, and overall metabolic flux. In contrast, Khlass showed higher concentrations of citric acid and citraconic acid, which may contribute to its distinct acidity profile and flavor characteristics. Mejhoul displayed comparatively lower levels of most organic acids, indicating a fading away of glycolysis or a more advanced stage of ripening (Figure 5).

### 2.5. Changes in Organic Acid Content

**Figure 6:**
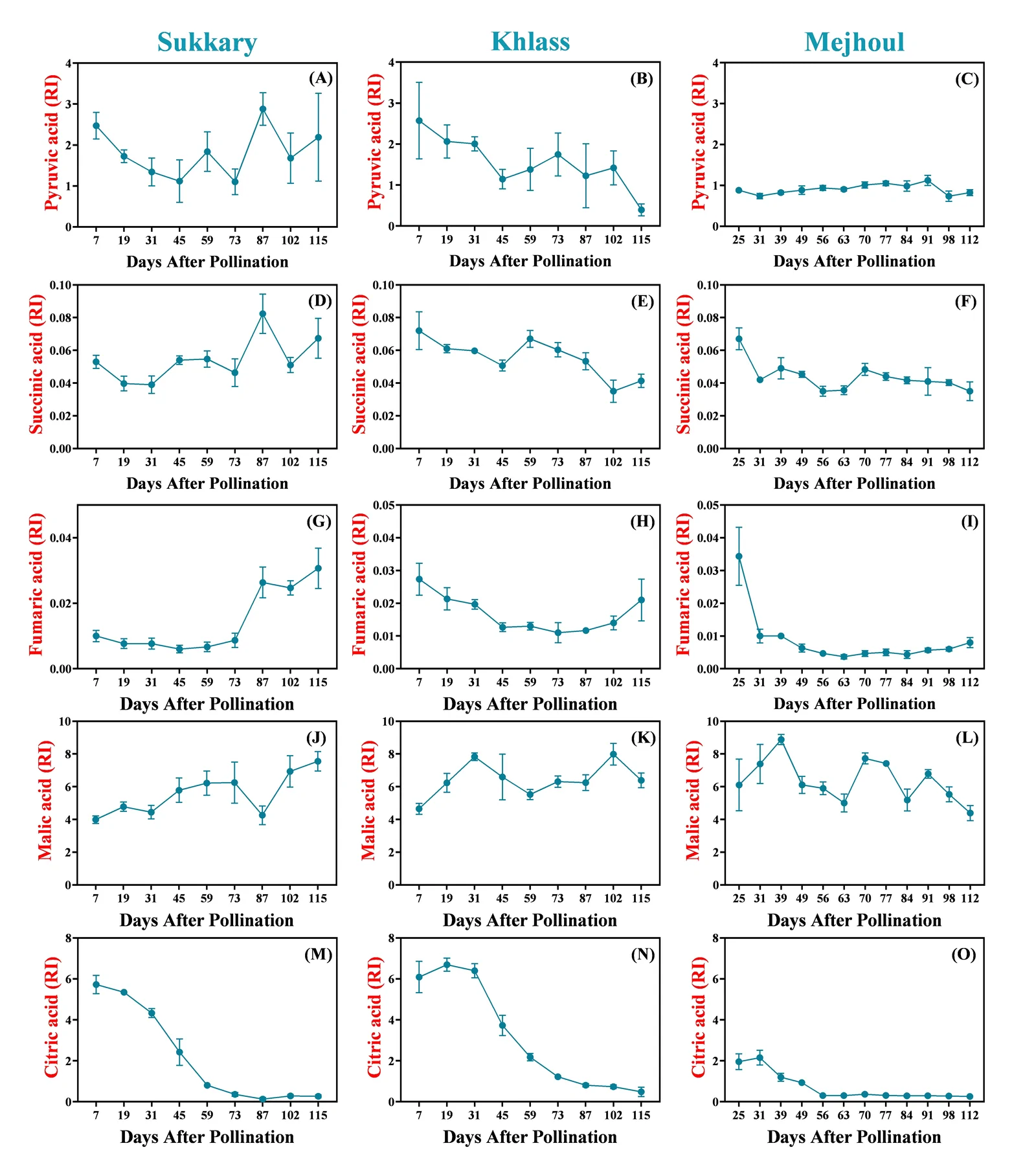
Changes in organic acid content in Sukkary, Khlass, and Mejhoul cultivars during fruit development. (**A**–**O**) depict the relative intensity of pyruvic, succinic, fumaric, malic, and citric acids over time.

The analysis of organic acid profiles revealed that date palm fruits are naturally rich in diverse organic acids, which vary in concentration depending on the cultivar and developmental stage. In Sukkary, the highest level of pyruvic acid was observed at 87 DAP, whereas the lowest was recorded at 73–75 DAP (Figure 6A). Khlass exhibited its peak pyruvic acid concentration early, at 7 DAP, and with a sharp decline by 115 DAP (Figure 6B). In Mejhoul, pyruvic acid peaked at 91 days after pollination (DAP) and declined to its lowest level by 98 DAP (Figure 6C). Among the three, Sukkary retained the highest pyruvic acid levels at the final observation point. For succinic acid, Sukkary showed the highest concentration at 87 DAP and dropped at 19 and 31 DAP (Figure 6D). Khlass displayed elevated succinic acid at 7 and 59 DAP, followed by a significant decrease by 102 DAP (Figure 6E). In Mejhoul, the highest concentration of succinic acid was observed at 25 DAP and the lowest at 56 DAP (Figure 6F). By the end of the monitoring period, Sukkary had the highest succinic acid content among all cultivars. Fumaric acid accumulation varied significantly across cultivars. Sukkary reached the highest fumaric acid content at 115 DAP and the lowest at 45 DAP (Figure 6G). In contrast, Khlass had its peak early at 7 DAP, with a marked reduction between 45 and 102 DAP (Figure 6H). In Mejhoul, concentration was measured at 25 DAP, while the lowest levels were between 63 and 84 DAP (Figure 6I). Mejhoul showed higher fumaric acid levels at maturity compared to Sukkary and Khlass at the beginning of observations, while Sukkary had the highest fumaric acid at the final observation. Regarding malic acid, Sukkary exhibited its highest malic acid concentration at 115 DAP, while the lowest was observed at 7 and 87 DAP (Figure 6J). In Khlass, the highest malic acid levels occurred at 102 DAP, and the lowest at 7 DAP (Figure 6K). Mejhoul peaked at 39 DAP and declined to its lowest point by 112 DAP (Figure 6L). At the final observation, Sukkary maintained higher malic acid levels than both Mejhoul and Khlass. Citric acid content showed a more variable trend. In Sukkary, the concentration was highest at 7 DAP and lowest at 87 DAP (Figure 6M). Khlass peaked at 19 and 31 DAP, while the lowest levels were observed at 115 DAP (Figure 6N). Mejhoul had the highest citric acid level at 31 DAP, with a significant decline between 56 and 112 DAP (Figure 6O). By the end of the observation period, citric acid levels were relatively similar among all three cultivars.

**Figure 7:**
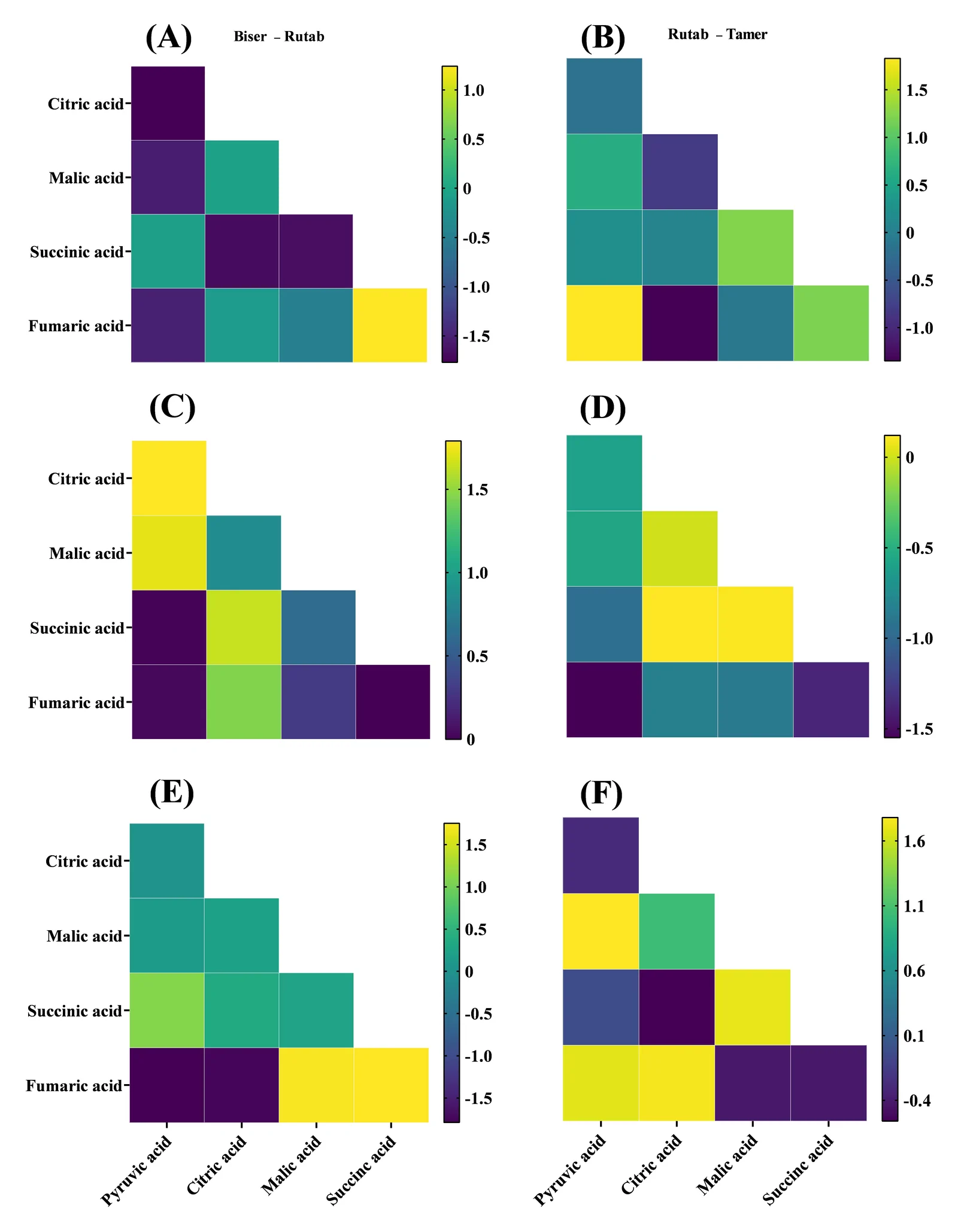
Changes in acid content of Sukkary (**A**,**B**), Khlass (**C**,**D**), and Mejhoul (**E**,**F**) cultivars at the Biser to Rutab and Rutab to Tamer stages.

The results demonstrate that organic acid metabolism is highly dynamic during date fruit maturation and differs among cultivars. During the Biser–Rutab transition, most cultivars exhibited increases in one or more TCA cycle intermediates, particularly malic and succinic acids, reflecting active respiratory metabolism associated with fruit growth. In contrast, the Rutab–Tamer transition was characterized by greater cultivar-specific changes. Sukkary showed a marked increase in pyruvic acid, Khlass accumulated malic and succinic acids, whereas Mejhoul exhibited increases in pyruvic, citric, and malic acids. Meanwhile, fumaric acid generally declined during ripening in Khlass and Mejhoul, suggesting its consumption as fruit metabolism shifted toward sugar accumulation (Figure 7).

### 2.6. Amino Acid Content

**Figure 8:**
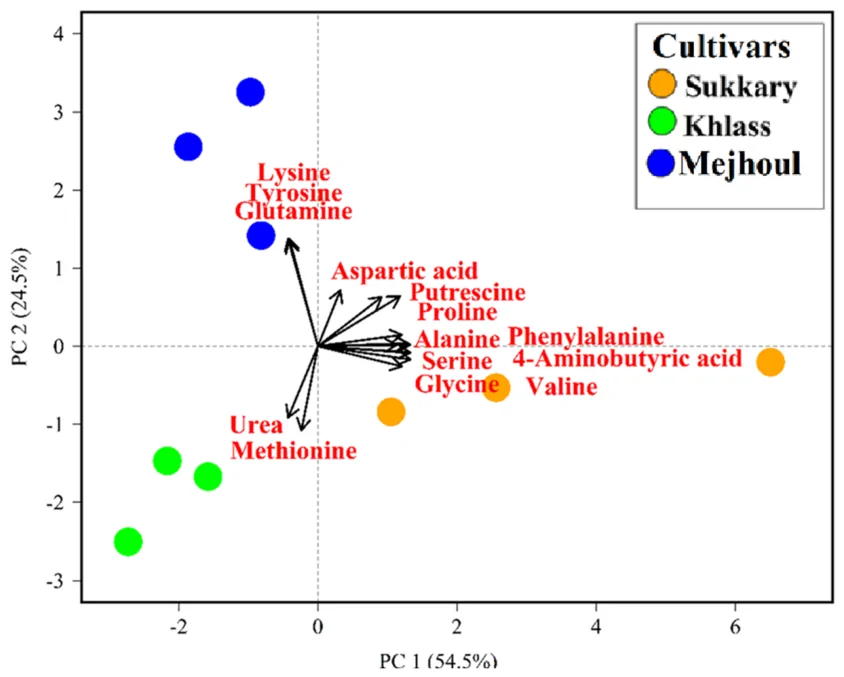
Principal component analysis (PCA) of amino acid profiles in Sukkary, Khlass, and Mejhoul date palm cultivars. The PCA reveals clear cultivar-specific clustering based on amino acid composition. Sukkary samples clustered distinctly due to high levels of aspartic acid, alanine, GABA, glycine, and valine. Mejhoul showed elevated levels of lysine, tyrosine, and glutamine, while Khlass was characterized by higher levels of methionine and urea. PC1 and PC2 account for 45.5% and 24.5% of total variance, respectively, highlighting the metabolic diversity among cultivars.

Principal component analysis (PCA) of the amino acid profiles revealed distinct clustering patterns among the three date palm cultivars, indicating cultivar-specific metabolic compositions (Figure 8). The Sukkary cultivar was characterized by higher concentrations of several amino acids, including aspartic acid, putrescine, alanine, phenylalanine, serine, 4-aminobutyric acid (GABA), glycine, and valine. In contrast, the Mejhoul cultivar exhibited elevated levels of lysine, tyrosine, and glutamine, while the Khlass cultivar was distinguished by higher levels of urea and methionine. These variations highlight the metabolic diversity among cultivars and may reflect differences in their physiological roles during fruit development. Glew et al. [39], for example, found that during the course of maturation of medlar fruit, lysine, tyrosine, and glutamate decrease.

### 2.7. Changes in Amino Acid Content

**Figure 9:**
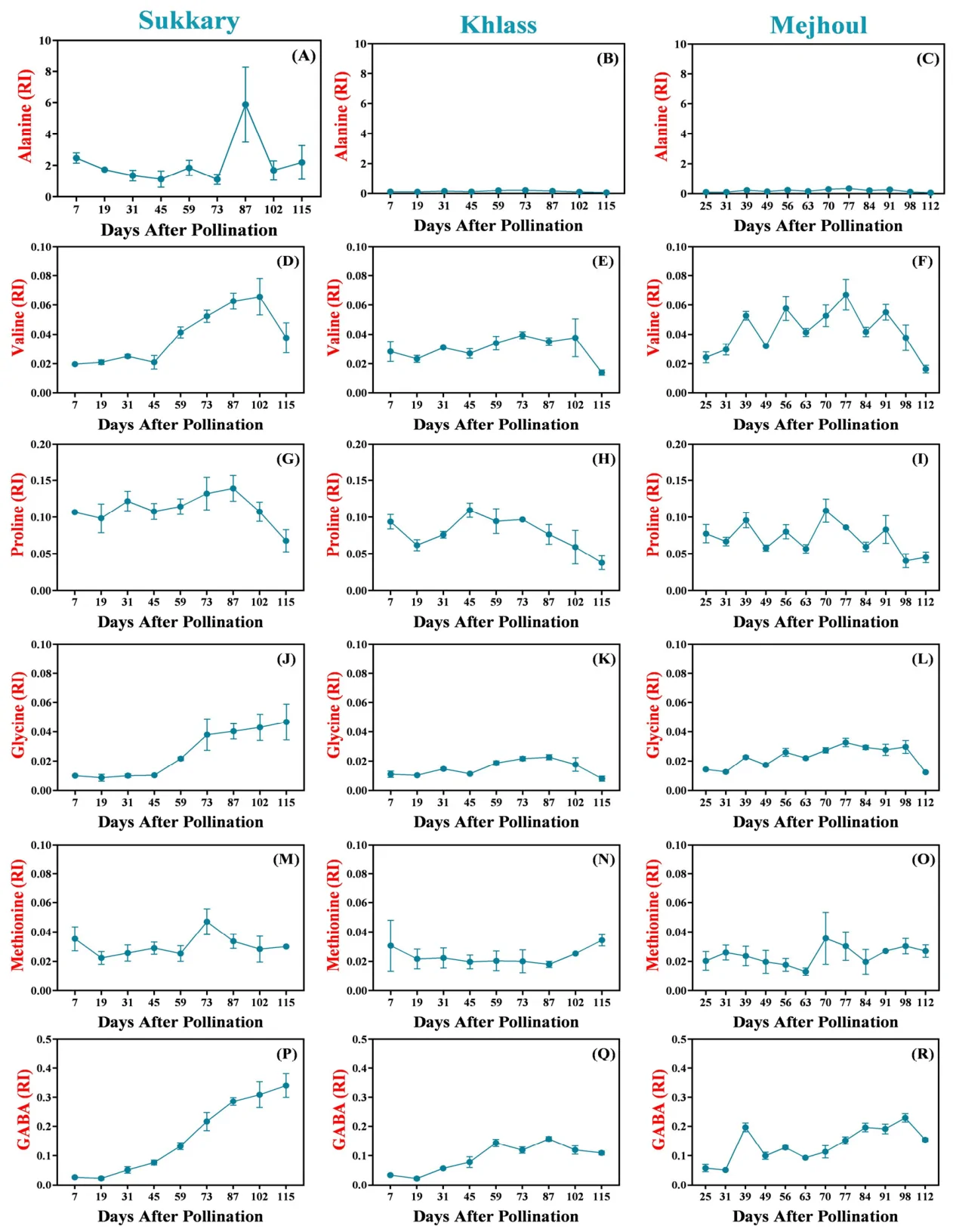
Temporal profiles of selected amino acids in Sukkary, Khlass, and Mejhoul date palm cultivars during fruit development. (**A**–**R**) show relative intensity (RI) changes in amino acids including alanine, valine, proline, glycine, methionine, and GABA over time.

The amino acid content of Sukkary and Khlass cultivars was assessed from 7 to 115 DAP, with sampling conducted at two-week intervals. Sukkary cultivar showed higher alanine (Figure 9A) content than the Khlass and Mejhoul cultivars (Figure 9B,C). For valine, a similar trend was observed for all three cultivars, where in Sukkary (Figure 9D) and Khlass (Figure 9E) cultivars the highest valine content was measured at 87 DAP and in Mejhoul at 77 DAP (Figure 9F). Sukkary had higher valine content compared to Khlass and Mejhoul. For proline, the highest amount was detected at 87 days after pollination in Sukkary (Figure 9G), 45 days after pollination in Khlass (Figure 9H), and 70 days after pollination in Mejhoul (Figure 9I), where the lowest proline content was observed at 115 DAP for Sukkary and Khlass and 98 DAP for Mejhoul. At the final observation, Sukkary had higher proline compared to Khlass and Mejhoul. For glycine, the highest amount was determined at 115 DAP in Sukkary (Figure 9J), 87 DAP in Khlass (Figure 9K), and 98 DAP in Mejhoul (Figure 9L), where the lowest glycine amount was determined at 45 DAP in Sukkary, 115 DAP in Khlass, and 112 DAP in Mejhoul (Figure 9J–L). At the final observation, Sukkary had a higher amount of glycine compared to Khlass and Mejhoul. For methionine, the highest amount was detected at 73 DAP in Sukkary, 115 DAP in Khlass, and 70 DAP in Mejhoul, where the lowest levels were determined at 19 DAP in Sukkary (Figure 9M), 87 DAP in Khlass (Figure 9N), and 63 DAP in Mejhoul (Figure 9O). At final observation, Sukkary and Khlass showed more methionine content compared to Mejhoul. For GABA (gamma-aminobutyric acid), the highest amount was detected at 115 DAP in Sukkary (Figure 9P), 87 DAP in Khlass (Figure 9Q), and 98 DAP in Mejhoul (Figure 9R), where the lowest was determined at 19 DAP in Sukkary and Khlass, and 35 DAP in Mejhoul. At final observation, Sukkary showed more GABA compared to Khlass and Mejhoul.

**Figure 10:**
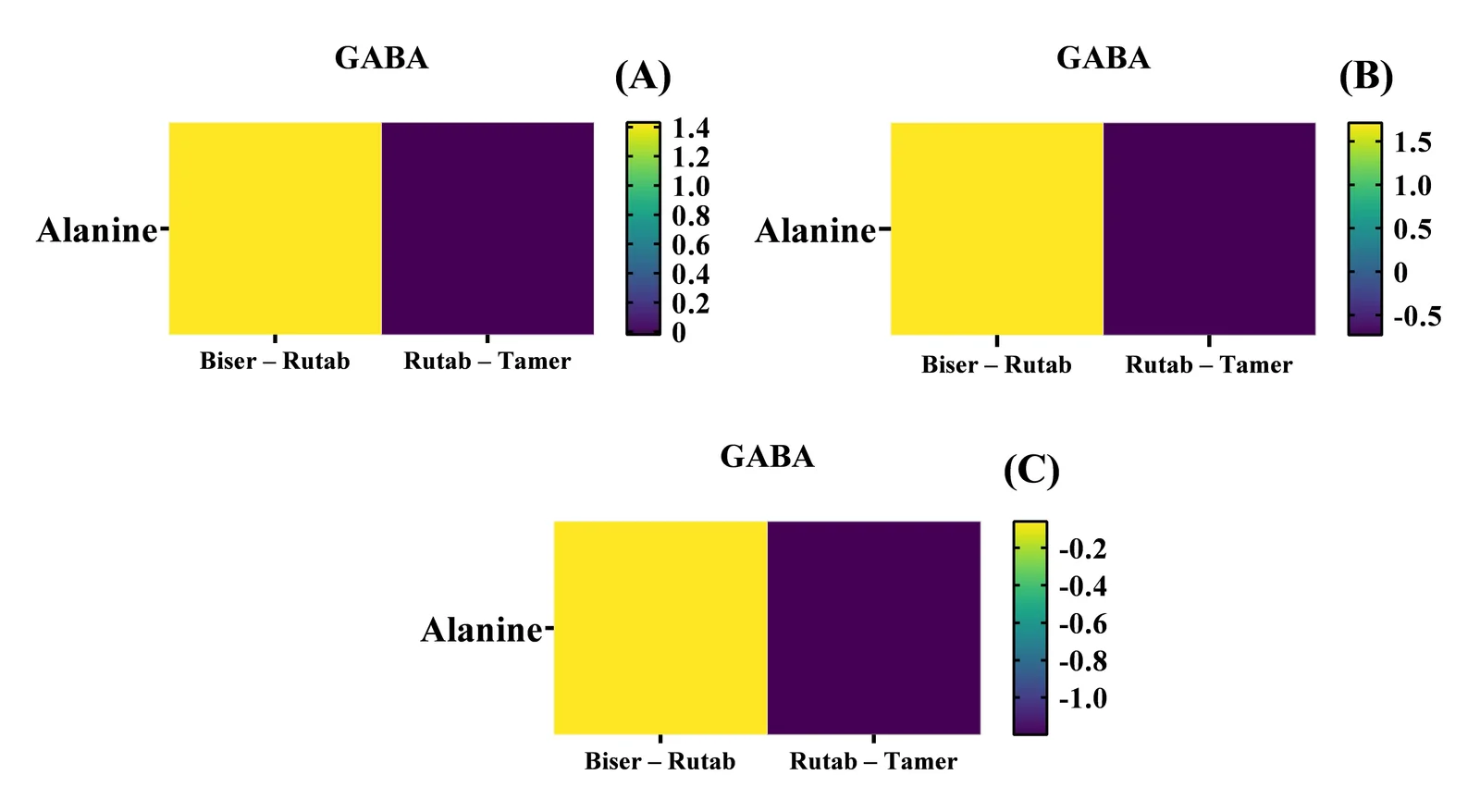
Changes in amino acid content of Sukkary (**A**), Khlass (**B**), and Mejhoul (**C**) cultivars at the Biser to Rutab, and Rutab to Tamer stages.

The results indicate that alanine and GABA accumulate during the early stages of fruit ripening but decline as fruits progress to the Tamer stage. The increase during the Biser–Rutab transition suggests that these amino acids play important roles in supporting active metabolism, nitrogen assimilation, and stress adaptation during fruit growth. Their subsequent decrease during the Rutab–Tamer transition indicates their utilization in metabolic pathways associated with fruit maturation (Figure 10).

### 2.8. Polyphenol Content

**Figure 11:**
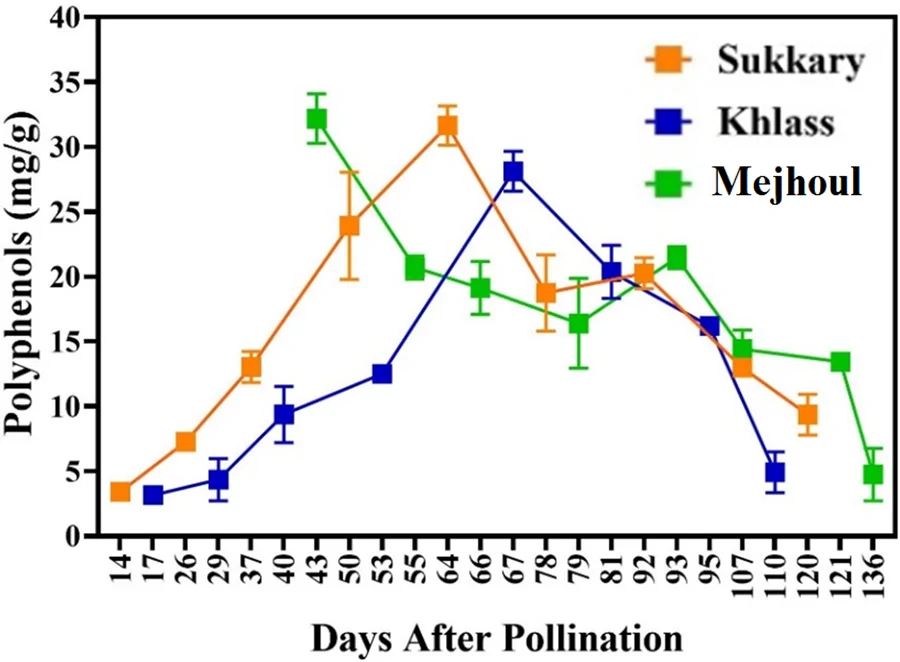
Polyphenol content in Sukkary, Khlass, and Mejhoul date palm cultivars at different developmental stages.

The analysis of polyphenol content revealed that Sukkary and Khlass cultivars exhibited peak levels at 64 and 66 days after pollination (DAP), respectively, whereas the maximum polyphenol concentration in Mejhoul was observed earlier, at 40 DAP. Notably, at 120 DAP, Sukkary maintained a higher polyphenol content compared to the other cultivars (Figure 11).

### 2.9. Total Metabolomic Profile

**Figure 12:**
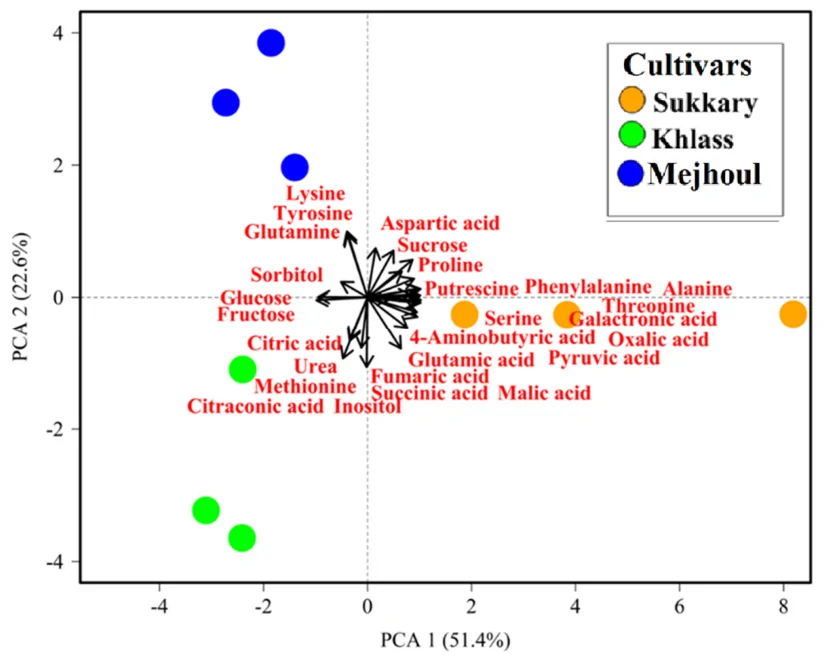
Principal component analysis (PCA) of total metabolomic profiles in Sukkary, Khlass, and Mejhoul date palm cultivars.

Principal component analysis (PCA) of the metabolomic profiles of Sukkary, Khlass, and Mejhoul cultivars revealed distinct clustering patterns, with PC1 accounting for 51.4% and PC2 for 22.6% of the total variance (Figure 12). The Sukkary cultivar was characterized by elevated levels of several amino acids, including aspartic acid, putrescine, alanine, phenylalanine, serine, 4-aminobutyric acid (GABA), glycine, and valine, suggesting a high degree of nitrogen metabolism and active biosynthesis of proteinogenic and signaling compounds. In contrast, Mejhoul exhibited higher concentrations of lysine, tyrosine, and glutamine, indicating a different amino acid metabolic profile. The Khlass cultivar stood out due to its higher levels of urea and methionine, which may reflect differences in nitrogen assimilation and sulfur-containing compound synthesis. Sukkary also demonstrated higher concentrations of key organic acids such as oxalic acid, pyruvic acid, fumaric acid, succinic acid, galacturonic acid, and malic acid. These metabolites are associated with the tricarboxylic acid (TCA) cycle and cell wall metabolism, indicating enhanced respiratory activity and cell wall remodeling processes, traits commonly linked to fruit maturation and metabolic flux. On the other hand, Khlass showed increased levels of citric acid and citraconic acid, compounds often associated with acidity and unique flavor attributes, possibly contributing to its distinct taste profile. Mejhoul presented comparatively lower concentrations of most organic acids, suggesting early deactivation of GAPDH (Glyceraldehyde-3-phosphate dehydrogenase) to stop glycolysis and/or enhanced gluconeogenesis with activated PEPCK (phosphoenolpyruvate carboxykinase), leading to glucose accumulation and decreased TCA levels.

### 2.10. Correlation Analysis

**Figure 13:**
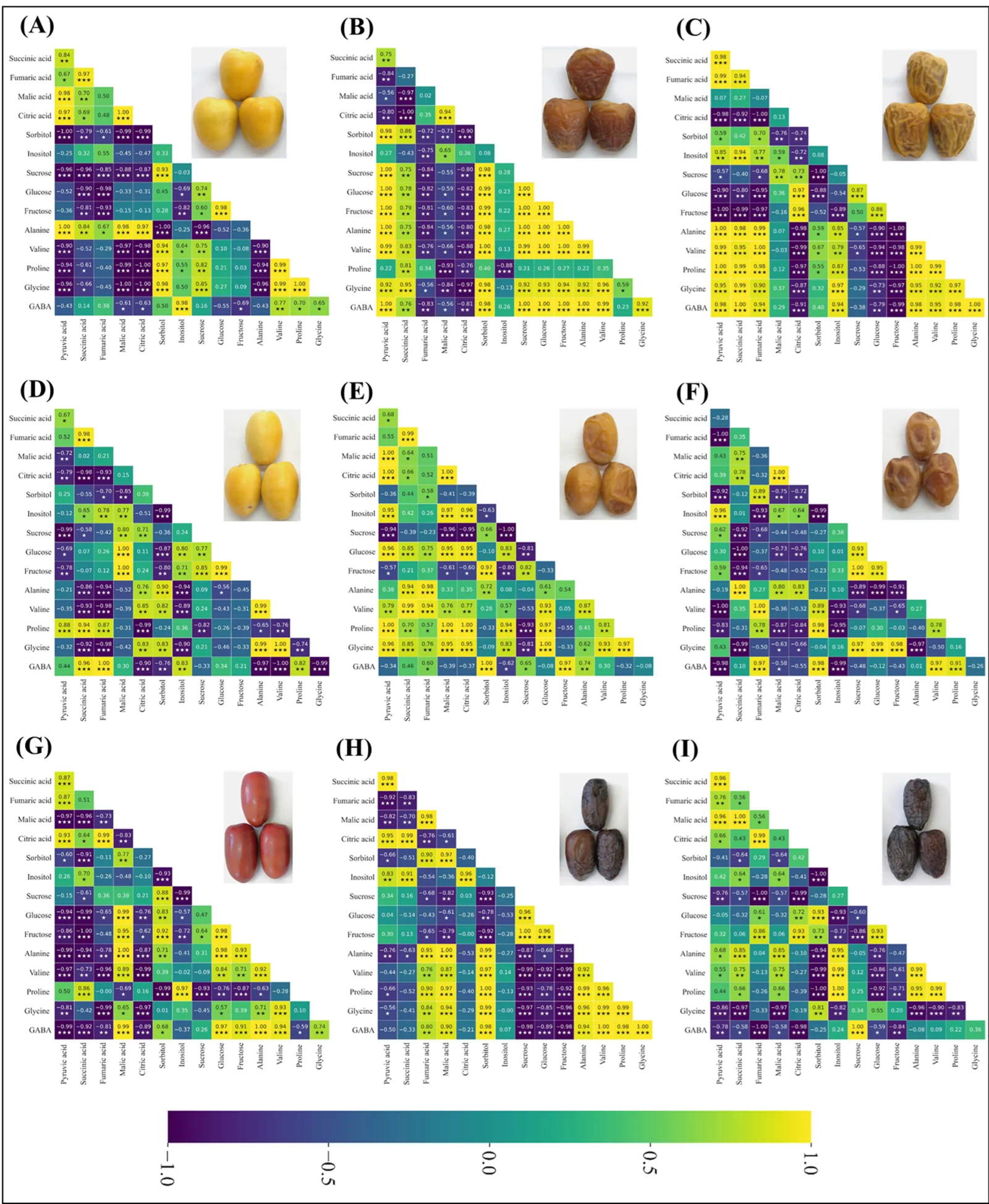
Correlation analysis of biochemical components in date fruits at three maturation stages for all three cultivars. Sukkary cultivar at the Biser (**A**), Rutab (**B**), and Tamer (**C**) stages; Khlass cultivar at the Biser (**D**), Rutab (**E**), and Tamer (**F**) stages; and Mejhoul cultivar at the Biser (**G**), Rutab (**H**), and Tamer (**I**) stages. Statistical significance is denoted as *** *p* < 0.001, ** *p* < 0.005, and * *p* < 0.01.

Correlation analysis demonstrated a clear shift in metabolite relationships during fruit maturation, reflecting substantial metabolic reprogramming from the Biser to the Tamer stage. At the Biser stage, strong positive correlations were mainly observed among organic acids, indicating coordinated organic acid metabolism during active fruit growth. For example, in Sukkary (A), pyruvic acid was highly correlated with malic acid (r = 0.98) and citric acid (r = 0.97), while sorbitol showed strong negative correlations with several organic acids (up to r = −0.99). Similar trends were observed in Khlass and Mejhoul. As fruits progressed to the Rutab stage, correlations among sugars and amino acids became stronger, whereas sucrose generally exhibited negative correlations with glucose and fructose, reflecting sucrose hydrolysis during ripening. In Sukkary (B), glucose and fructose showed an almost perfect positive correlation (r = 1.00), while sucrose was negatively correlated with glucose (r = −0.82). At the Tamer stage, sugars and amino acids formed highly interconnected networks, with glucose, fructose, alanine, valine, proline, and glycine showing consistently strong positive correlations (generally r > 0.95). In contrast, many organic acids displayed weaker or negative correlations with sugars, indicating their depletion as ripening progressed. Although these developmental trends were common across all three cultivars, Sukkary exhibited the strongest positive associations among sugars and amino acids, whereas Khlass and Mejhoul showed more variable metabolite interactions. These findings highlight the dynamic transition from an organic acid-dominated metabolism in immature fruits to a sugar- and amino acid-dominated metabolism in fully ripened fruits, with genotype-specific differences influencing biochemical composition during fruit maturation (Figure 13).

## 3. Discussion

The metabolomic profiles of Sukkary, Mejhoul, and Khlass date palm cultivars revealed significant biochemical diversity in terms of soluble solids, polyphenols, amino acids, sugars, and organic acids. These differences reflect cultivar-specific metabolic pathways and postharvest characteristics, which influence not only taste and texture but also nutritional and functional properties.

### 3.1. Total Soluble Solids (Brix)

The Brix (%) values were highest in Mejhoul, followed by Khlass and then Sukkary, indicating varying levels of sugar accumulation during fruit ripening (Figure 1). High Brix (%) in Mejhoul aligns with findings by Alam et al. [31], who reported Brix (%) values around 20.36 for soft date cultivars, correlating with advanced ripening and invert sugar accumulation. Khlass, a semi-soft cultivar, typically exhibits moderate Brix (%) levels in its syrup, whereas Sukkary, known for its dryness, often shows similar Brix (%) in its syrup, with 72.2 in Sukkary and 72.6 in Khlass, and there was no significant difference between them [40]. The international type of Mejhoul cultivar was also reported to have a higher concentration of total sugars, varying from 71 to 79 $g\times 10^{-2}\;g\;DW$ [41]. In the Barhi cultivar of date palm, the total soluble solids are reported to be 43.7–47.6% [42]. The differences in Brix (%) values also influence sensory attributes. Higher Brix (%) contributes to greater sweetness intensity, particularly in cultivars dominated by invert sugars like Mejhoul, while Sukkary achieves sweetness through high sucrose content, despite its comparatively lower total soluble solids. This distinction underscores the importance of sugar composition alongside Brix (%) in determining palatability and consumer preference. However, the total sugar content can be influenced by various factors, including environmental conditions and other biochemical compounds. We also investigated the correlations among biochemical components (Figure 13). The results suggest that organic acids may play a key role in determining the levels of total soluble sugars, although this might need further experimentation.

### 3.2. Sugar Composition

Sugar composition varied significantly among the cultivars. Sukkary was characterized by a dominance of sucrose, while Mejhoul and Khlass contained higher levels of glucose and fructose. This differentiation corresponds to the classification of date fruits into sucrose-type and invert sugar-type cultivars, as described by Zhang C. R. et al. [43], who classified Deglet Noor, Sukkary Al Qassim, and Nabtat Ali date cultivars as sucrose-type. Sucrose accumulation in Sukkary indicates limited invertase activity during ripening, a trait common in dry cultivars that retain stored disaccharides. In contrast, Mejhoul and Khlass undergo active sucrose hydrolysis via invertase, leading to an increase in monosaccharides such as glucose and fructose. These sugars contribute not only to the sweetness intensity but also to faster glycemic responses [44,45]. The presence of fructose may modulate the glycemic index [46]. Inositol accumulation patterns observed in this study varied notably among the three cultivars, with Mejhoul peaking at 90 DAP, Sukkary at 87 DAP, and Khlass at 102 DAP, highlighting genotype-specific temporal regulation during fruit development. These findings align with previous research on date palm and other fruit species, where inositol content has been shown to increase during mid-development stages and decline toward full maturity. For instance, Thurston et al. [47] reported that in date fruits, inositol tends to accumulate during the active cell division and expansion phases, correlating with roles in osmoregulation and membrane biogenesis. Similarly, Loewus and Murthy [48] emphasized the significance of myo-inositol in early fruit growth due to its involvement in phytic acid biosynthesis and cell wall formation. In grapes, Carlos et al. [49] documented a comparable trend, where inositol peaked before véraison and decreased thereafter, mirroring the decline seen in Mejhoul and Khlass at later stages (112 DAP and beyond). The markedly higher early inositol levels in Sukkary and Khlass suggest a possible cultivar-specific metabolic prioritization, potentially linked to faster early developmental progression or stress adaptation. These findings reinforce the idea that inositol metabolism is not only developmentally regulated but also genetically influenced, supporting the hypothesis that different date palm cultivars possess unique sugar biosynthetic and transport mechanisms. This sugar dynamic also reflects underlying shifts in carbohydrate metabolism governed by glycolysis and gluconeogenesis. In cultivars where glucose and fructose dominate, glycolytic flux intensifies to support the high energy demands of ripening and flavor compound biosynthesis. Conversely, cultivars retaining high sucrose levels may utilize gluconeogenic enzymes to modulate sugar pools more conservatively. These processes not only determine sweetness but also influence metabolic health impacts and suitability for processing. The sugar profile is also critical in industrial processing, as high monosaccharide content enhances fermentability and flavor complexity [50], while sucrose-rich dates offer longer shelf life and less hygroscopicity, beneficial for packaging and storage. The results demonstrate that sugar metabolism differs among date palm cultivars during fruit ripening. All three cultivars showed an increase in reducing sugars during maturation, consistent with the enzymatic conversion of sucrose into glucose and fructose. However, the dominant sugar varied among cultivars.

### 3.3. Organic Acids

Organic acid analysis revealed that Sukkary contained higher levels of malic and citric acids compared to Mejhoul and Khlass. Importantly, the acids used to differentiate the samples into distinct groups were present in relatively significant concentrations across all examined cultivars, indicating their fundamental role in date fruit metabolism. A positive relationship was observed between organic acids and amino acids, suggesting coordinated metabolic activity, while a significant negative relationship emerged between organic acids and sugars, consistent with the progressive conversion of organic acids into sugars during fruit ripening. These patterns are further illustrated in Figure 13.

Alahyane et al. [51] reported that date fruits generally contain a high level of organic acids. Ghnimi et al. [52] compared 21 Emirati date cultivars and observed a higher concentration of organic acids in Mejhoul. Kamal-Eldin et al. [53] noted that soft and semi-soft date cultivars typically exhibit elevated sugars and organic acids, emphasizing that these acids are key intermediates in the tricarboxylic acid cycle and contribute to pH regulation, microbial resistance, and flavor development. Malic acid, the predominant organic acid in Mejhoul, imparts mild sourness and enhances flavor complexity, while citric acid improves antioxidant buffering capacity. Similarly, Martín-Sánchez et al. [54] reported that malic acid, as the main organic acid, is abundant in Spanish date cultivars during the Khalal and Rutab stages.

The relatively low concentration of organic acids in Mejhoul and Khlass aligns with its dry texture and high sugar-to-acid ratio, which contributes to its sweeter, milder flavor preferred in certain markets [55]. As noted by Ghnimi et al. [13], cultivars with high acid content may possess enhanced preservation properties, whereas low-acid cultivars such as Sukkary are more prone to spoilage under poor storage conditions, despite being more appealing to consumers who prefer less acidic fruits.

Figure 13 shows that metabolic alterations are most dynamic at the Rutab stage across all cultivars, with pronounced differences compared to the Biser and Tamer stages. During the Biser stage, relatively minor differences are observed among the three cultivars. In contrast, clear varietal distinctions emerge at the Rutab stage. In the Sukkary cultivar, amino acid metabolism appears particularly active, as indicated by the presence of numerous yellow and dark boxes, especially at the Rutab and Tamer stages. Overall, metabolic activity is markedly altered in all cultivars during the Rutab stage, reflected by the abundance of yellow and dark boxes. Conversely, the Biser and Tamer stages exhibit comparatively more stable metabolic profiles, characterized by a higher proportion of green boxes.

### 3.4. Amino Acids

A higher abundance of essential and non-essential amino acids was found in Sukkary, particularly valine (7–102 DAP), glutamine, and arginine. These amino acids are crucial for protein synthesis, nitrogen metabolism, and stress responses [56]. Hamad et al. [32] reported that the Khlass cultivar has higher amino acids, while the comparison was not done with Sukkary and Mejhoul. Different date cultivars with reduced sugar metabolism often retain more amino acids due to preserved protein turnover pathways and less carbon reallocation towards saccharide synthesis; however, sometimes it can be parallel [57]. Glutamine and arginine, in particular, are indicators of nitrogen assimilation [58] and osmotic adjustment under dry conditions [59]. Their elevated presence in Sukkary aligns with observations by Abdelbaky et al. [60], who noted higher concentrations of these amino acids in the Sukkary cultivar compared to Ajwa, which is grown in Saudi Arabia, and the Siwi cultivar, which is grown in Egypt. By contrast, Mejhoul and Khlass showed lower amino acid concentrations, suggesting decreased glycolytic activity and resource reallocation toward sugar and organic acid synthesis, as proposed by [61] and Salomón-Torres et al. [62] who observed low amino acids in seeds of Deglet Noor, Khadrawy, Mejhoul, and Zahidi cultivars. The amino acid profile has implications for nutritional value, particularly for diets lacking in animal protein, and offers potential for value-added functional food development.

**Figure 14:**
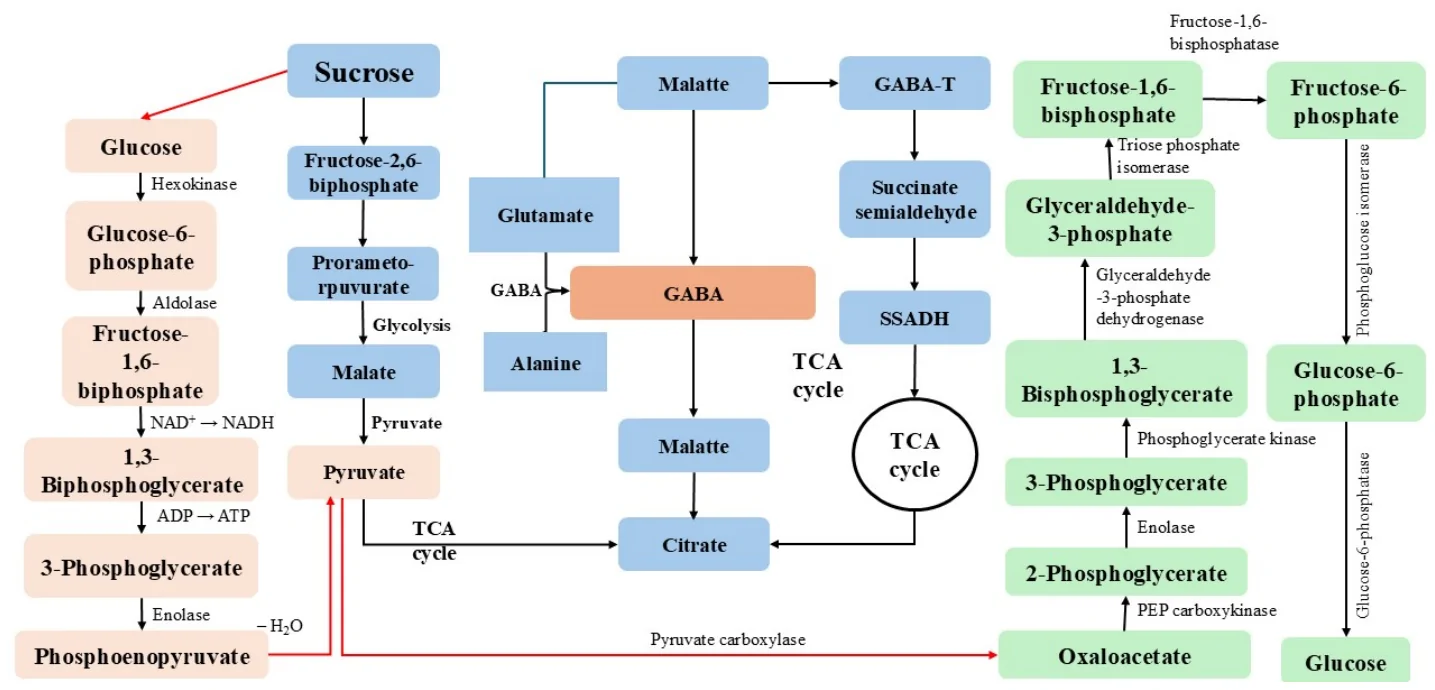
Integrated view of key metabolic pathways contributing to fruit ripening and quality in date palm cultivars. The schematic outlines glycolysis (light red), GABA biosynthesis (light blue), and gluconeogenesis (light green) pathways. These processes influence sugar accumulation, acid degradation, and stress signaling, which collectively shape fruit flavor, texture, and nutritional value during development.

Furthermore, the maturation of date fruits involves a tightly regulated coordination between glycolysis and gluconeogenesis pathways. Glycolysis provides energy and metabolic intermediates necessary for ripening, particularly in soft cultivars like Mejhoul and Khlass. These cultivars exhibit elevated levels of glycolytic enzymes that promote the conversion of glucose and fructose into pyruvate and ATP, fueling biosynthetic pathways (Figure 14) related to cell wall softening and flavor formation. In contrast, in dry cultivars such as Sukkary, gluconeogenic activity may help maintain sugar homeostasis by regenerating glucose from non-carbohydrate sources, particularly amino acids like glutamine and alanine. The presence of key enzymes such as phosphoenolpyruvate carboxykinase (PEPCK) and fructose-1,6-bisphosphatase during later ripening stages has been reported in other fruit crops and may indicate a similar metabolic adaptation in date palms. This metabolic shift is essential for sustaining sugar levels under water-limited or low-energy conditions, enhancing the nutritional quality and storage resilience of dry-type cultivars.

In addition to these amino acids, γ-aminobutyric acid (GABA) also plays a critical role in plant carbon and nitrogen metabolism, acting as a metabolic hub at the intersection of these pathways [63,64]. GABA is produced through the GABA shunt, which bypasses two steps of the TCA cycle and contributes to energy balance under stress and during ripening. Although not quantified in the current study, its potential role in date palm fruit ripening merits attention. In other species like *Camellia sinensis* (tea plant), exogenous application of *trans*-2-hexenal influenced the expression of genes such as *CsGAD* and *CsGABAT* (involved in GABA biosynthesis) as well as *CsCHS* and *CsF3H*, which are crucial for flavonoid metabolism [65,66]. Notably, anthocyanin concentrations and GABA showed a positive correlation, indicating GABA’s indirect influence on pigmentation and antioxidant capacity. It is conceivable that similar regulatory cross-talk exists in date palms, particularly affecting polyphenol biosynthesis pathways in cultivars like Sukkary, which exhibits high polyphenol content. Further investigation into the presence and regulation of the GABA shunt and its interaction with secondary metabolite pathways in date fruits could illuminate new mechanisms governing fruit quality and stress adaptation.

Moreover, the dynamic shifts in metabolite concentrations during maturation may reflect stage-specific metabolic regulation involving the tricarboxylic acid (TCA) cycle and its peripheral pathways. Studies on fruit maturation have identified a distinct switch in carbohydrate metabolism during the transition from early development to ripening, marked by differential expression of enzymes like phosphoenolpyruvate carboxylase (PEPC2) and phosphoenolpyruvate carboxykinase (PEPCK). This shift suggests a re-routing of carbon flux, with early-stage metabolism favoring biosynthesis and ripening stages supporting respiration and energy release. In this context, GABA plays a transitional role: during the onset of ripening, it is first metabolized into malate via succinate semialdehyde, and then routed through pyruvate, eventually re-entering the TCA cycle as citrate (Figure 14). This not only links GABA metabolism to respiration but also indicates that GABA serves as a buffer for carbon skeleton redistribution during the energy-intensive ripening process [67]. These insights suggest that the metabolic plasticity observed in cultivars like Sukkary, which display elevated amino acids and organic acids, could reflect enhanced GABA shunt activity and more efficient coordination of primary and secondary metabolism.

### 3.5. Polyphenolic Content

Among the three cultivars, Sukkary exhibited the highest total polyphenol content, followed by Khlass and Mejhoul (Figure 2). These findings correspond to those of Saleh et al. [68], who demonstrated that the Sukkary cultivar retains elevated levels of phenolic compounds even at full ripeness, followed by Khlass. Ouamnina et al. [69] also highlighted the strong correlation between phenolic content and antioxidant activity in Moroccan date cultivars. It has also been reported that the Tunisian cultivar of date has higher polyphenolic content [70]. The moderate polyphenol content observed in Sukkary suggests preservation of antioxidant components due to lower enzymatic degradation and reduced moisture levels, characteristics typical of dry-type dates [71]. Conversely, Mejhoul displayed the lowest phenolic content among the three, consistent with findings by Salomón-Torres et al. [72], who noted that the pulp of Mejhoul, which was cultivated in Mexico, contained 1.6 mg GAE/100 g DW, which reveals that this cultivar tends to lose phenolic compounds due to oxidative enzymatic activity and dilution by water accumulation during late ripening. Such variation in polyphenol content influences not only antioxidant potential but also shelf-life and resistance to microbial spoilage, as polyphenols play protective roles against oxidation and microbial degradation.

Sukkary showed the highest levels of both polyphenols and the aromatic amino acid phenylalanine, suggesting a strong link between primary and secondary metabolism in this cultivar. Phenylalanine serves as the key precursor for the phenylpropanoid pathway, which leads to the synthesis of a wide range of polyphenolic compounds, including anthocyanins, flavonoids, tannins, and lignins. This metabolic relationship indicates that the elevated phenylalanine levels in Sukkary likely contribute to its superior polyphenol profile, enhancing its antioxidant capacity and potential health benefits. Further transcriptomic or enzymatic studies could investigate whether enzymes such as phenylalanine ammonia-lyase (PAL) are more active in this cultivar, catalyzing the first committed step in polyphenol biosynthesis.

## 4. Materials and Methods

### 4.1. Sample Preparation

This study was carried out in Nafeesa farm of YALA Company, Shihya, Saudi Arabia. The plant material consisted of 6-year-old date palm (*Phoenix dactylifera* L.) trees of the cultivars Sukkary, Khlass, and Mejhoul, cultivated on the farm. The samples were collected directly from the company’s agricultural fields and were therefore not obtained from wild populations. The study was fully financed by YALA Company, and because the plant material was collected from their own cultivated trees, no external permits or governmental permissions were required for sample collection. The formal identification of the plant material was conducted by specialists from YALA Company at Nafeesa Farm, who confirmed the identity of the cultivars Sukkary, Khlass, and Mejhoul. To ensure traceability and future reference, voucher specimens of these date palm cultivars can be deposited in a recognized herbarium or institutional collection, and accession numbers will be provided upon deposition.

**Figure 15:**
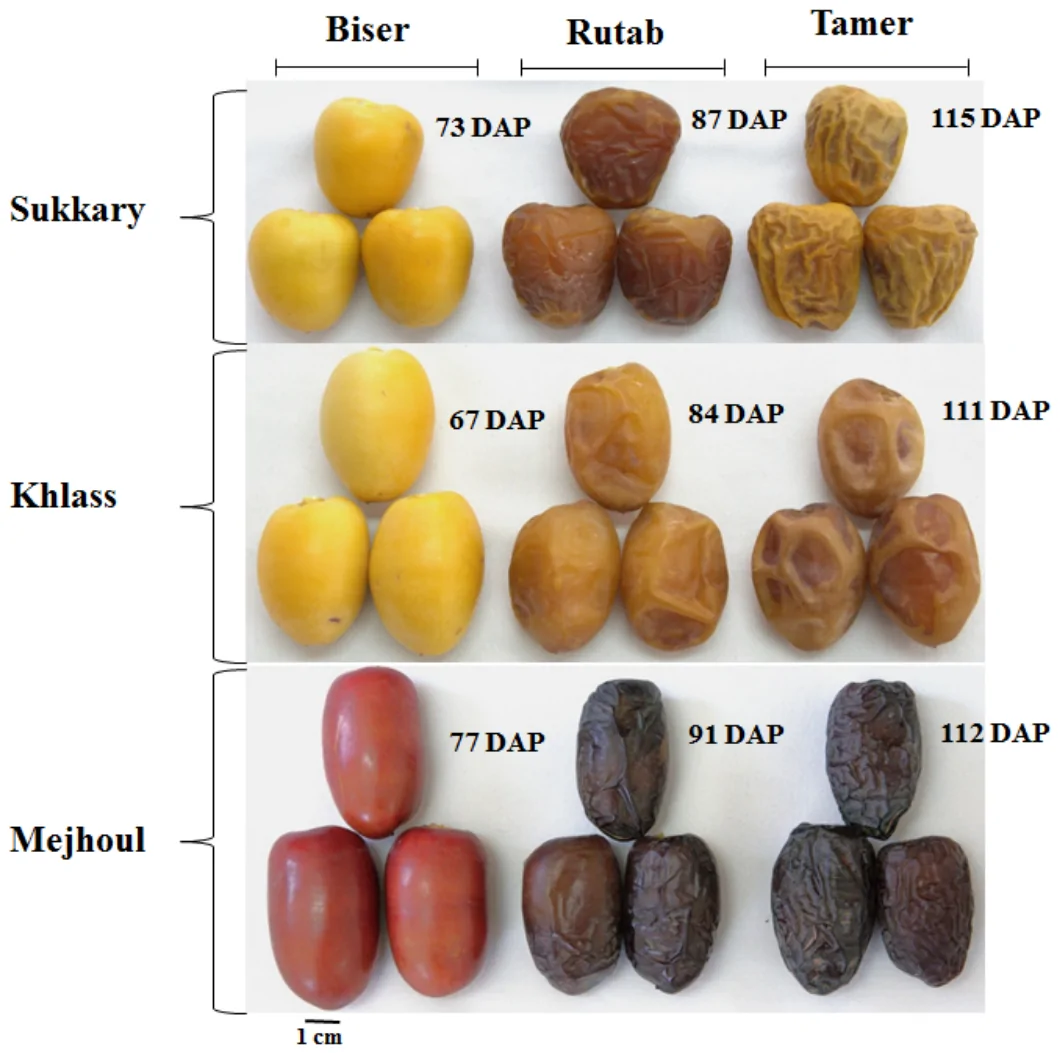
Developmental stages of date palm fruit ripening from Biser to Rutab to Tamer. This visual timeline contextualizes the physiological and metabolic changes occurring throughout fruit maturation. Biser (early), Rutab (semi-ripe), and Tamer (fully ripe) stages correspond to shifts in sugar content, acidity, polyphenols, and amino acids, as described in this study.

To monitor fruit development and biochemical changes, fruit sampling was carried out at regular intervals of approximately one to two weeks, following established protocols [73,74]. The ripening process was divided into three distinct stages to capture the progression from immature to fully ripe fruits. The first stage, Biser, occurred around 120 days after pollination (DAP), when the fruits began to change color and soften slightly. This was followed by the Rutab stage, at approximately 135 DAP, characterized by more pronounced softening and an increase in sweetness. Finally, by around 150 DAP, the fruits reached the Tamer stage, representing full ripeness with maximum sugar accumulation and reduced moisture content (Figure 15). Sampling strategies accounted for cultivar-specific differences in maturation. For the ‘Mejhoul’ cultivar, fruits were sampled up to 150 DAP due to their longer ripening period; however, the observed changes were not significant; therefore, 115 DAP was considered the tamer stage in Mejhoul, while for the earlier-maturing ‘Sukkary’ and ‘Khlass’ cultivars, sampling was completed by 115 DAP. Three trees were selected out of 27 for each cultivar, and three fruit clusters on each tree were pollinated with the same pollen source to maintain consistency. At each sampling interval, one strand was collected from each cluster, and a fruit was harvested from the middle position of each strand. This approach yielded three fruits per tree, or a total of nine fruits per cultivar for each sampling time. The fruit maturation stages occurred at different times among the three cultivars. The Bisser stage was reached at 73 DAP in Sukkary, 67 DAP in Khlass, and 77 DAP in Mejhoul. The Rutab stage occurred at 87 DAP in Sukkary, 84 DAP in Khlass, and 91 DAP in Mejhoul. Finally, the Tamer stage was reached at 115 DAP in Sukkary, 111 DAP in Khlass, and 112 DAP in Mejhoul (Figure 15).

To prepare the fruits for biochemical analysis, the nine collected fruits were pooled and mixed thoroughly. They were then ground into a fine powder to ensure homogeneity. From this powdered mixture, three representative samples were prepared, each comprising three fruits, and subsequently analyzed for their biochemical composition using gas chromatography–mass spectrometry (GC-MS). This approach allowed accurate and reproducible profiling of primary and secondary metabolites.

All plant research was conducted in strict compliance with institutional, national, and international guidelines, specifically those of Tokyo University of Agriculture. Fieldwork was performed on cultivated farm trees owned by YALA Company, adhering to local legislation in Saudi Arabia. Since the plant material was obtained from company-owned sources, no additional permissions or licenses were required.

### 4.2. Brix Measurements

On each sampling day, six dates were collected from three different trees. Of these, juice was extracted from three fruits to determine Brix, and its analysis was conducted at the Laboratory of YALA in Qassim, Saudi Arabia. Brix was measured using the method previously described [75,76]. For the measurement of TSS (Brix), all the date fruits harvested at the same stage and almost the same size were sliced and crushed to make juice at 4 °C. After filtration using two layers of mesh cloth and homogenization, a refractometer was used for the measurement of the Brix levels (%).

### 4.3. Measuring Metabolome

For GC-MS sample preparation, seeds were removed, and the fruit flesh was rapidly frozen in liquid nitrogen. Samples were then freeze-dried and ground into a fine powder using a mortar and pestle under cold conditions to preserve metabolite stability, and the samples were moved to the Laboratory of Tropical Horticulture Science, Tokyo University of Agriculture. The metabolome was measured using GC-MS following the method previously described [77,78,79]. To investigate the metabolites of date fruits, samples were collected from three cultivars: Mejhoul, Sukkary, and Khlass. From a population of 29 trees for each variety, three trees were randomly selected. On each selected tree, three fruit clusters were pollinated using the same pollen source (male flower) to ensure uniformity. At the time of sampling, one strand was cut from each of the three fruit clusters, and one fruit was collected from the middle position of each strand. Consequently, three fruits were collected from each tree, resulting in a total of nine fruits per variety at each sampling time. Whole date fruits were homogenized in a mortar pre-cooled with liquid nitrogen. A total of 100 mg of homogenized date fruit powder was used to prepare each sample. Each sample was combined with one zirconia bead and 250 µL of methanol, then mixed thoroughly in a mixer mill MM400 for 2 min at 27 Hz. Subsequently, 250 µL of chloroform was added, and the samples were incubated in a thermomixer for 3 min at 37 °C and 1200 rpm. Next, 50 µL of standard solution and 125 µL of ultrapure water were added to each sample, which was centrifuged at 1500× *g* for 10 min at 25 °C. Careful extraction of 80 µL of the supernatant was transferred to a 1.5 mL Eppendorf tube and evaporated for 2 h. Samples were then placed in a freeze dryer and stored overnight. Following lyophilization, 40 µL of methoxamine solution was added to each sample, which was incubated in a thermomixer for 90 min at 37 °C. Subsequently, 50 µL of N-methyl-N-trimethylsilyl trifluoroacetamide (MSTFA) was added, and samples were incubated for an additional 30 min under the same conditions. A 50 µL aliquot of the final solution was used for GC-MS analysis.

For the preparation of reagents, ribitol was used to prepare the standard solution by dissolving 0.2 mg of ribitol in 1 mL of ultrapure water. The methoxamine solution was prepared by dissolving 20 mg of methoxamine in 1 mL of pyridine. Metabolomic analysis was performed using a gas chromatography–mass spectrometry (GC-MS) system (SHIMADZU GC-2010 (Shimadzu Corporation, Kyoto, Japan) coupled with GCMS-QP2010 Plus). A DB column (0.25 mm internal diameter, 30 m length, 1.00 µm film thickness, Agilent Technologies, Santa Clara, CA, USA) was used. The GC conditions were as follows: inlet temperature of 280 °C, split injection at 10:1, oven temperature initially held at 60 °C for 1 min, ramped to 320 °C at 4 °C/min, and held at 320 °C for 10 min, with a helium flow rate of 1.1 mL/min. MS conditions included scanning mode, a transfer line temperature of 290 °C, and an ion source at 200 °C. Mass spectra were recorded at a scan rate of 1 scan/s with a mass-to-charge ratio (*m*/*z*) range of 45–600.

Data analysis was conducted using the multivariate analysis software Pirouette version 5.0 (Infometrix, Inc., Bothell, WA, USA).

### 4.4. Polyphenolic Measurements

On each sampling day, six dates were collected from three different trees. Juice was extracted from three of the fruits to determine total phenolic content, which was analyzed at the Laboratory of YALA in Qassim, Saudi Arabia, using the method previously described [80].

### 4.5. Statistical Analysis

The statistical analysis was conducted using R software version 4.1.2. Significant differences between the treatment groups were determined using a one-way analysis of variance (ANOVA), with a significance threshold set at *p* < 0.05. Additionally, principal component analysis (PCA) was utilized to analyze metabolome data, providing a comprehensive overview of the key metabolites that contributed to the observed variations. All the graphs were plotted using GraphPad Prism (v11.0.0).

## 5. Conclusions

The findings revealed significant biochemical variability among the Sukkary, Mejhoul, and Khlass date palm cultivars, highlighting their unique metabolomic signatures. Mejhoul exhibited the highest total soluble solids, with Brix (%) values increasing steadily and reaching their maximum at 120 DAP, consistently surpassing Sukkary and Khlass. These elevated Brix (%) levels correspond to substantially higher glucose and fructose accumulation, particularly near maturity—when fructose and glucose reached their peak values at approximately 150 and 91 DAP, respectively. In contrast, Sukkary was distinguished by markedly higher sucrose levels, which continued to rise until 115 DAP and remained substantially higher than in Mejhoul and Khlass. Mejhoul also showed sorbitol concentrations approximately 1.5-fold higher than Khlass and 1.9-fold higher than Sukkary, underscoring key differences in carbon allocation and sugar metabolism. Organic acid profiles also revealed strong differentiation. Sukkary exhibited higher levels of malic, succinic, fumaric, oxalic, and pyruvic acids, indicating sustained TCA cycle activity and cell wall metabolism toward maturity. In contrast, Khlass showed higher citric and citraconic acid levels, contributing to its acidity, while Mejhoul generally exhibited lower concentrations of most organic acids by late maturity—suggesting reduced glycolytic flux and a shift toward gluconeogenesis and sugar accumulation. Amino acid profiling further distinguished the cultivars. Sukkary consistently showed higher levels of alanine, valine, proline, glycine, and GABA, particularly at maturity, whereas Mejhoul accumulated more lysine, tyrosine, and glutamine. Khlass was uniquely characterized by elevated methionine and urea. PCA of the amino acid dataset explained 70% of the total variance, with clear clustering that reflects cultivar-specific nitrogen metabolism and ripening physiology. Collectively, these findings demonstrate that Sukkary is a high-phenolic, sucrose-rich cultivar with strong nitrogen and organic acid metabolism; Khlass exhibits distinctive sulfur- and citric-acid-related metabolic features; and Mejhoul is characterized by high Brix (%) values and late-stage accumulation of invert sugars with reduced organic acid content. The combination of quantitative metabolite profiling and PCA-based clustering provides a robust framework for differentiating the three cultivars and highlights the metabolic drivers underlying their sensory qualities, nutritional attributes, and potential functional properties. This detailed biochemical characterization supports the development of cultivar-specific strategies for harvest optimization, quality assessment, and targeted utilization in food and nutraceutical applications.

## Data Availability

The original contributions presented in this study are included in the article. Further inquiries can be directed to the corresponding author.

## Abbreviations

GCC	The Gulf Cooperation Council
GC	Gas Chromatography
TCA	Tricarboxylic Acid
GABA	Gamma-Aminobutyric Acid
